# Metagenome Sequences of a Thermophilic Anaerobic Digester Adapted to a Low C/N Ratio, High-Ammonia Feedstock (Poultry Litter)

**DOI:** 10.1128/genomeA.00598-18

**Published:** 2018-06-21

**Authors:** David H. Huber, Jesus E. Chavarria-Palma, Sridhar A. Malkaram, Natalia A. Montenegro-Garcia, Vadesse Lhilhi Noundou, Ifeoma R. Ugwuanyi, Teodoro Espinosa-Solares

**Affiliations:** a Department of Biology, West Virginia State University, Institute, West Virginia, USA; b Gus R. Douglass Institute, West Virginia State University, Institute, West Virginia, USA; c Departmento de Ingeniería Agroindustrial, Universidad Autónoma Chapingo, Chapingo, Mexico

## Abstract

We sequenced the metagenome of a pilot-scale thermophilic digester with long-term, stable performance on poultry litter feedstock which has a very low C/N ratio, a high ammonia level, and high lignocellulose content. Firmicutes were the dominant phylum (68.9%). Other abundant phyla included Bacteroidetes, Euryarchaeota, and Thermotogae. This microbiome represents a hydrogenotrophic methanogenic community with high diversity.

## Abstract (precis)

We sequenced the metagenome of a pilot-scale thermophilic digester with long-term, stable performance on poultry litter feedstock which has a very low C/N ratio, a high ammonia level, and high lignocellulose content. Firmicutes were the dominant phylum (68.9%).

## GENOME ANNOUNCEMENT

Anaerobic digestion (AD) is used to treat concentrated organic wastes and to simultaneously produce bioenergy (methane). The methanogenic food web in digesters can be described as consisting of three trophic levels where heterogeneous substrates are converted into biogas. These are hydrolysis/primary fermentation, secondary fermentation (including syntrophy), and methanogenesis. AD systems differ in design and applications, particularly with regard to substrate and temperature, which introduces considerable phylogenetic and metabolic diversity (1–3). The high temperature of thermophilic AD destroys pathogens and increases substrate solubility and reaction rates. However, the food web may be less stable and more easily disturbed by substrate changes than mesophilic AD (4). Recent metagenomic analysis has shown many new bacterial species and higher-level clades in AD food webs (3, 5).

We have operated a pilot-scale thermophilic digester that was stabilized on poultry litter substrate. This substrate has a very low C/N ratio (6 to 7), which is far below the reported optimum of 20 to 30 (6). In addition, this feedstock produces high ammonia levels which can be inhibitory, and it has high lignocellulose content. This particular microbiome has shown long-term stability toward this suboptimal substrate. Several studies from our group have examined important features of this digester, including mass-energy balance (7), feed-loading frequency (8), and power consumption efficiency (9). Bacterial diversity of this microbiome was previously analyzed with 16S rRNA gene-targeted pyrosequencing (10); adaptability during codigestion was also studied (11).

The digester is a 40-m^3^, thermophilic (56°C), continuously stirred tank reactor (CSTR) with mixing provided by recirculation of headspace gas. The design was described in Espinosa-Solares et al. (7). In the current study, the digester had stable performance with the same substrate for about 7 years. At the time of sampling, digester performance variables during a 4-week period were the following: methane, 60.1 ± 1.6%; biogas, 6.6 ± 0.9 m^3^ d^−1^; pH, 7.9 ± 0.1; N-ammonia, 1,846.9 ± 180.3 mg L^−1^; volatile acids, 4,173.7 ± 1,135.1 mg L^−1^; and chemical oxygen demand, 31,631.4 ± 8,612.8 mg L^−1^. A liquid sample was collected from a port in the middle of the reactor. The liquid was centrifuged at 20,000 rpm for 30 min at 4°C to pellet solids. The pellet was stored at −80°C. DNA was extracted using a PowerSoil DNA isolation kit (MoBio Laboratories). DNA was sequenced at Carver Biotechnology Center, University of Illinois, with the Roche 454 GS FLX Titanium pyrosequencing system. The metagenome consists of 1,248,442 reads and 642.5 million total bases. The average read length is 405 bp. Taxonomic classification was done using the MG-RAST server (12) employing the M5NR database (13). The most abundant phyla are Firmicutes (68.9%), Bacteroidetes (7.9%), Euryarchaeota (6.1%), Actinobacteria (4.6%), Proteobacteria (3.9%), Thermotogae (3.4%), and Synergistetes (1.5%). Twenty methanogen genera were identified. The most abundant methanogens are Methanoculleus (82%) and Methanothermobacter (9%), which utilize hydrogenotrophic methanogenesis. Functional analysis showed high levels of carbohydrate metabolism, particularly mono-, di-, and oligosaccharides. This microbiome is a model for thermophilic bioenergy conversion processes.

### Accession number(s).

The metagenome sequences were deposited in the NCBI database in the Sequence Read Archive under accession number SRP139605.

## References

[1] Zhang W, Werner JJ, Agler MT, Angenent LT 2014 Substrate type drives variation in reactor microbiomes of anaerobic digesters. Bioresour Technol 151:397–401. doi:10.1016/j.biortech.2013.10.004. 24183494

[2] Nelson MC, Morrison M, Yu Z 2011 A meta-analysis of the microbial diversity observed in anaerobic digesters. Bioresour Technol 102:3730–3739. doi:10.1016/j.biortech.2010.11.119. 21194932

[3] Treu L, Kougias PG, Campanaro S, Bassani I, Angelidaki I 2016 Deeper insight into the structure of the anaerobic digestion microbial community; the biogas microbiome database is expanded with 157 new genomes. Bioresour Technol 216:260–266. doi:10.1016/j.biortech.2016.05.081. 27243603

[4] Labatut RA, Angenent LT, Norman RS 2014 Conventional mesophilic vs. thermophilic anaerobic digestion: a trade-off between performance and stability? Water Res 53:249–258. doi:10.1016/j.watres.2014.01.035. 24530545

[5] Campanaro S, Treu L, Kougias PG, De Francisci D, Valle G, Angelidaki I 2016 Metagenomic analysis and functional characterization of the biogas microbiome using high throughput shotgun sequencing and a novel binning strategy. Biotechnol Biofuels 9:26. doi:10.1186/s13068-016-0441-1. 26839589 PMC4736482

[6] Speece RE 1996 Anaerobic biotechnology for industrial wastewaters. Archae Press, Nashville, TN. 10.1021/es00115a725 22656942

[7] Espinosa-Solares T, Bombardiere J, Chatfield M, Domaschko M, Easter M, Stafford D, Castillo-Angeles N, Castellanos-Hernandez N 2006 Macroscopic mass and energy balance of a pilot plant anaerobic bioreactor operated under thermophilic conditions. Appl Biochem Biotechnol 129–132:959–968. doi:10.1385/ABAB:132:1:959. 16915704

[8] Bombardiere J, Espinosa-Solares T, Domaschko M, Chatfield JM 2007 Thermophilic anaerobic digester performance under different feed-loading frequency. Appl Biochem Biotechnol 137-140:765–775. doi:10.1007/s12010-007-9096-5. 18478433

[9] Espinosa-Solares T, Valle-Guadarrama S, Bombardiere J, Domaschko M, Easter M 2009 Effect of heating strategy on power consumption and performance of a pilot plant anaerobic digester. Appl Biochem Biotechnol 156:35–44. doi:10.1007/s12010-008-8487-6. 19127441

[10] Smith AM, Sharma D, Lappin-Scott H, Burton S, Huber DH 2014 Microbial community structure of a pilot-scale thermophilic anaerobic digester treating poultry litter. Appl Microbiol Biotechnol 98:2321–2334. doi:10.1007/s00253-013-5144-y. 23989973

[11] Sharma D, Espinosa-Solares T, Huber DH 2013 Thermophilic anaerobic co-digestion of poultry litter and thin stillage. Bioresour Technol 136:251–256. doi:10.1016/j.biortech.2013.03.005. 23567688

[12] Keegan KP, Glass EM, Meyer F 2016 MG-RAST, a metagenomics service for analysis of microbial community structure and function. Methods Mol Biol 1399:207–233. doi:10.1007/978-1-4939-3369-3_13. 26791506

[13] Wilke A, Harrison T, Wilkening J, Field D, Glass EM, Kyrpides N, Mavrommatis K, Meyer F 2012 The M5nr: a novel non-redundant database containing protein sequences and annotations from multiple sources and associated tools. BMC Bioinformatics 13:141. doi:10.1186/1471-2105-13-141. 22720753 PMC3410781

